# Tackling land’s ‘stubborn materiality’: the interplay of imaginaries, data and digital technologies within farmland assetization

**DOI:** 10.1007/s10460-023-10453-3

**Published:** 2023-05-01

**Authors:** Sarah Ruth Sippel

**Affiliations:** Geography Department, University of Münster, Heisenbergstr. 2, 48149 Münster, Germany

**Keywords:** Farmland investment, Assetization, Land imaginaries, Digital data, Digital technologies, Digital farming

## Abstract

The nature of farming is – still – an essentially biological, and thus volatile, system, which poses substantial challenges to its integration into financialized capitalism. Financial investors often seek stability and predictability of returns that are hardly compatible with agriculture – but which are increasingly seen as achievable through data and digital farming technologies. This paper investigates how farmland investment brokers engage with, perceive, and produce farming data for their investors within a co-constructive process. Tackling land’s ‘stubborn materiality’ for investment, I argue, has material and immaterial components: it includes the re-imagination of farming as a financial asset that delivers reliable income streams for investors; and the re-engineering of farmland’s concrete materialities with digital farming technologies. Farmland investment brokers develop investor-suitable farmland imaginaries, underpinned by storytelling as well as the calculative ‘evidence’ of (digital) data. At the same time, digital technologies have become a key tool for transforming farms into ‘investment grade assets’ endowed with the rich data on farm performance and financial returns requested by investors. I conclude that the assetization and digitization of farmland need to be seen as closely intertwined and mutually reinforcing processes and identify key areas for future research on this intersection.

## Vignette

In the spring of 2016, I sat with David^1^ in a coffee shop in a suburb of Sydney. At that time, David worked for a small farmland investment company that offered professional management services to ‘lower-end’ private direct investors, i.e. investors who want to invest below 20 million dollars. One of the services his company provided was ‘due diligence’ – an appraisal of the productive capacity of the property the client is interested in, based on climatic conditions, the pasture, the soil resources, and the topography of the property. Based on these factors, the client receives an estimation of the economic value of the property’s production under the ‘right’ management conditions. This due diligence, David explained, also entailed an assessment of the risks that could affect the asset. ‘What factors do you include in your risk assessment,’ I asked him, ‘and what are the most important risks?’ ‘Well,’ he responded, ‘it starts with the climate, and then extends right through to soil and pastures, and how those resources are being managed and maintained. Because everything in agriculture is changeable. Every day things are changing. It’s a biological system!’ Agriculture, he went on, is a system in a constant state of flux, where every management decision has an effect on multiple parts of the system. ‘It’s not like a factory’, he insisted, ‘that exists largely in isolation from the rest of the environment. Compared to a factory, you have limited control over a farm. You can influence things, but there are very few things that you can control in an absolute sense.’

## Introduction

Over the past few decades, the finance sector has shown a rising and continued interest in ‘all things nature’ (Loftus and March 2015; Ouma et al. 2018). Originally sparked by the global food price, fuel, and financial crises in 2007/08, the promotion of agri-food as a worthwhile investment opportunity has recently persisted through yet another crisis, the COVID-19 pandemic, which agri-food investors have used to further strengthen the case for agri-investment (Fairbairn and Guthman 2020; Reisman 2021). While the 2007/08 conjunction of events represented the ‘initial’ crisis moment that incentivized investors to search for alternative investment possibilities, the pandemic has been touted as having consolidated agriculture as an alternative investment class (Sippel 2022). However, as critical scholarship on farmland investments has argued, farmland does not lend itself easily to financial investment (see, e.g., Fairbairn 2020; Goldstein and Yates 2017; Li 2014; Ouma 2016, 2020a; Pedersen and Buur 2016). There are various hurdles to farmland investment, as land is considered a resource unlike any other. Due to land’s multiple ontologies, its life-giving affordances, its strong association with national territory, and the multiplicity of moral and emotional connotations associated with land, land is a particularly challenging and contested investment object (Li 2014; Sippel and Visser 2021). This holds notably true for settler state contexts such as Australia, where current systems of land control have been built upon the (continued) dispossession of lands and the denial of the rights of Indigenous peoples, while land has simultaneously played a crucial role within settler state nation-building processes (Dussart and Poirier 2017; Liboiron 2021; Wolfe 2006). Adding to these complex, social, cultural, and emotional relationships with land, the very nature of farming itself also presents an obstacle to farmland investment. As my informant David pointed out, farming is – still – an essentially biological, and thus volatile and largely unpredictable, system. More than forty years after Mann and Dickinson (1978) formulated their seminal thoughts on the incompatibilities between agriculture and the requirements of capitalist production, biological factors and the ‘stubborn materiality’ of farming are still posing substantial challenges to farmland’s integration into today’s increasingly financialized capitalism. Financial investors are looking for a stability and predictability in returns, which is hardly compatible with the manifold natural vagaries of farming David described in the introductory vignette.

Located within the farmland investment literature, this paper specifically addresses the material barriers that farmland presents to its financial assetization. Scholarship on farmland investment has only recently started to address the material dimensions within the financialization of farmland (Sippel and Visser 2021). Fairbairn, for instance, identified a range of strategies – from portfolio diversification to the construction of commensurative metrics to digital data – used by investors to deal with the material obstacles to farmland investment (Fairbairn 2020). Visser (2021) and Böhme (2021) further demonstrated that if overly optimistic land imaginaries, based on environmental narratives and marketing strategies, ignore, or clash with, the concrete physical and climatic realities of farmland localities, the outcome can be farm failures, bankruptcy, and environmental damage rather than lucrative returns for investors. Adding to this literature, this paper focuses specifically on the role of farmland investment brokers and the strategies these brokers apply, when faced with the ‘inconvenient materiality’ of farming (Fairbairn 2020, p. 81).

Tackling land’s materiality and biological messiness for investment, I argue, involves two processes, one material and one immaterial: the re-imagining of farming as a financial asset class, delivering reliable income streams for investors; and the re-engineering of farms’ concrete materialities with digital farming technologies on the ground. I demonstrate how farmland investment brokers co-create investor-suitable farmland imaginaries, which depict farming as a controllable activity that has calculable and predictable risks. These farmland imaginaries are underpinned by storytelling as well as by the ‘evidence’ of (digital) data, statistics, and the modeling of future farm outputs, which help sustain the imaginary of calculable nature. To this aim, I investigate how farmland investment brokers engage with, perceive, and produce both imaginative and number-based representations of farming for their investors within a process that I consider a form of ‘co-production’ (Jasanoff 2004) of farming knowledge between farmland investment brokers and financial investors.^2^

Moreover, I show that digital farming technologies – or what has been termed precision agriculture, ‘smart farming’, or agriculture 4.0 – are considered the key tool to endow farms with the one crucial asset they increasingly and inevitably need to become ‘investment grade assets’: data. As Duncan et al. (2022, p. 242) have recently argued, digital farming technologies facilitate a certain way of ‘seeing’ in agriculture, namely ‘one that is built on market logic and rationality and leads to an algorithmic governmentality and framework for “making sense” of land as an asset’. Farms are thus increasingly equipped with new ‘data infrastructures’ (Goldstein and Nost 2022) of ‘smart’ technologies that produce the kind of farm data financial investors are looking for to make farming ‘legible’ to them – that is to say calculable for risk-return assessments and commensurable with other assets. Putting these data infrastructures in place includes a material transformation of farms, from installing sensors to the use of remote devices to autonomous vehicles. Placing the emphasis on the role of data and digital farming technologies within farmland investments, this paper ultimately points to the intersection between two major transformations of agri-food, the financialization and increasing digitization of farming, and joins recent scholarship in suggesting that these two are taking place as closely interlinked and mutually reinforcing processes within the forging of agri-food futures (Duncan et al. 2022; Fairbairn 2020; Ouma 2020a).

This paper presents empirical material stemming from just over one year of fieldwork conducted on the intersections between finance and farming in Australia between 2016 and 2019. Within the universe of global farmland investments, Australia holds a particular place. Financial investors have targeted Australia for its assumed stable political system, its commitment to the neo-liberal principles of business orientation, open markets, and foreign direct investments, well-developed infrastructure, comparatively ‘undervalued’ land prices, proximity to Asian markets, and counter-seasonality to investments in the northern hemisphere, amongst others.^3^ My research included participant observation at industry events and at the community and farm levels, as well as more than 70 interviews with various actors in the Australian ‘agri-finance’ space. In this paper, I predominantly draw on some 25 interviews with representatives of farmland investment companies, or, as I will call them, ‘farmland investment brokers’.

As intermediaries between the worlds of ‘global finance’ and ‘local farms’, these farmland investment brokers usually worked for farmland investment companies in a leading position, or, in some cases, set up these companies themselves. These farmland investment companies undertook and facilitated financial capital investments in farmland and farming on behalf of their clients, who were mostly either private investors (i.e. ‘high net-worth individuals’ and family offices) or institutional investors (e.g. pension and endowment funds). The investment profile of the companies ranged from smaller, ‘boutique’ companies that specialized in clients investing within the range of 10–20 million dollars to companies focused on institutional capital with several hundreds of millions of dollars of assets under management. Investments took place in a variety of productions, from broad acre cropping to tree plantations (such as almonds) to cattle breeding and dairy, and mostly followed the ‘own-operate’ model of farmland investment (where land and farm are both owned by, and operated on behalf of, the investor).

My informants were involved in a variety of farmland investment activities, from raising capital, to the development of customized investment models, to the placement of capital into concrete farms on the ground, to the management of farms to make sure these farms deliver the requested returns. All these activities require important ‘translational skills’ in order to present farming to investors in the language of investment, which, as I will show below, first and foremost means a language that persuades investors of the controllability and calculability of farming’s risks and returns. Given that my informants often had a background in both (family) farming and finance, they were in a suitable position to make these translations necessary to channel capital from financial markets into farming operations on the ground.

In what follows, I will first sketch out the analytical framework for my analysis, drawing on the recent literature on assetization and farmland imaginaries as well as insights from social studies of finance and critical data studies. This is followed by a presentation and discussion of my empirical findings on how farmland investment brokers ‘co-produce’ farmland imaginaries suitable for investment. I conclude by identifying questions for further research at the intersection of assetization and digitization of agri-food.

## Rendering land investable: farmland assetization, land imaginaries, and critical data studies

Cautioning against linear presentations of ever-increasing land commodification, financialization, and dispossession, scholars have emphasized that rendering land investable relies on a host of complex activities that require further investigation (see, e.g., Goldstein and Yates 2017; Le Billon and Sommerville 2017; Ouma 2016, 2020a; Pedersen and Buur 2016). From a holistic understanding, land’s ‘investability’ not only pertains to the possibility to invest in land, as such, but also the multitude of parameters within which investments are socially embedded and which stakeholders factor in while assessing potential investments (Sippel and Weldon 2021, p. 309). Amongst other factors, such as governmental and regulatory actions or sociopolitical, cultural, and moral considerations, these parameters also include standard investment criteria, such as compliance with risk assessments and rates of return. Recent scholarship on farmland assetization, land imaginaries, and insights from social studies of finance and critical data studies are useful to investigate how these parameters are transferred to farming to increase land’s investability.

The notion of ‘assetization’ as a key operation within the broader process of financialization (Birch and Ward 2022),^4^ is helpful to better understand how nature and society are becoming increasingly incorporated into present-day financialized capitalism (Langley 2020; see also Ducastel and Anseeuw 2017; Ouma 2020b; Visser 2017). Based on, but distinct from, commodification, ‘assetization’ pertains to the transformation of things into tradable and recurring sources of revenue (Birch 2017). In comparison to a commodity – which is characterized by its exchangeability at a specific moment in time (Appadurai 1986) – an asset is distinct in delivering a consistent and reliable income stream (Birch and Muniesa 2020). Visser (2017) further suggests that five requirements need to be fulfilled for the construction, transformation, and framing of something as an ‘asset’ that can be recognized by financial markets: the object (or service) (1) has to have the potential to generate profit; (2) needs to be (considered as) scarce; (3) has a certain ‘liquidity’; (4) can be standardized in order to be comparable with other assets; and (5) its treatment as an asset, as such, needs to be perceived as legitimate. Next to questions surrounding the legitimacy of treating farmland as a financial asset (see Kish and Fairbairn 2018; Sippel 2018; Ouma 2020b), farmland’s low liquidity, poor standardization, and its lack of comparability with other asset classes, have represented major hurdles for its financial assetization. As Ouma (2016, p. 85; referring to Sherrick et al. 2013) pointed out, there is no ‘ticker’ for farmland investments, resulting in limited calculative commensurability of farmland with other asset classes.

To examine the ‘assetization work’ of farmland brokers within their efforts of rendering land investable, it is useful to investigate the financial practices, theories, and instruments they introduce and transfer to farmland and farming activities (Chiapello 2020). From this perspective, rendering land investable can be understood as the reconfiguration of farming as a particular type of financial space through a ‘shifting set of practices, logics and devices’ (Williams 2014, p. 402). Given the interest of this paper in the hurdles that material and biological vagaries and uncertainties represent to land’s investability, I will focus in particular on those sets of practices, logics, and devices that the finance sector has developed surrounding ‘risk’ as a central category in financial markets (Besedovsky 2018; de Goede 2004; Power 2014), and how these are being translated to farming.

Identifying, calculating, and selling risk sits at the heart of modern financial markets (de Goede 2004). Financial actors engage with the idea of risk in their everyday practices to describe possible futures and to increase the calculability of future events (Besedovsky 2018, p. 241). The meaning of risk, however, is not fixed, but is the result of contingent practices, which differ substantially in terms of how risk is conceived and measured, and how much trust is put into calculative models of risk assessment. Abstract ideas such as risk, as Besedovsky writes, thus only become ‘real’ through their performance in practice. These calculative practices and the specific conceptions of risk they entail can be seen as co-constitutive: ‘[c]alculative practices create the objects they measure and are at the same time the concrete manifestations of the abstract concepts they represent’ (Besedovsky 2018, p. 242). As a consequence, those who perform calculative risk practices also decide (to a certain extent) about the de facto meaning of what they are calculating, measuring, or evaluating. Hence, researching the role of risk within farmland investments means to understand the calculative practices surrounding risk in practice, and how changing risk practices (co-)produce new farmland imaginaries.

One crucial conundrum within the calculation of risk is the engagement with the radical uncertainty of the future, and the fact that calculations of the future can inevitably only take place based on past data. Beckert and Bronk (2018, p. 10) suggest that in situations where the future is not already ‘given’ and cannot be assumed to just replicate the past, actors resort to imagination and create imaginaries of the future in the form of ‘fictional expectations’. Such fictional expectations can be produced through the use of stories and narrations, which play an important role in finance and economic processes (see, e.g., Komporozos-Athanasiou and Fotaki 2020; Tarim 2012; Tsing 2000) as well as calculative devices, such as business plans or models. Resembling literary fiction in many ways (Vint 2019), fictional expectations are both constitutive and performative, that is to say they have real-world consequences as they affect the future by inspiring actions to bring about (or prevent) those futures envisaged in expectations (Beckert and Bronk 2018; Birch 2023). Fictional expectations surrounding land can thus be seen as attempts at calculating and predicting a certain farm future, while at the same time helping to performatively create this future.

The fictional expectations produced by farmland investment brokers as part of the finance and technology driven transformations of farmland can furthermore be usefully understood as containing and expressing specific ‘land imaginaries’. Drawing on scholarship engaging environmental, sociotechnical, and spatial imaginaries, the notion of land imaginaries pertains to the various ideas and societal understandings of what land is, and what it can, or should, do in society (Sippel and Visser 2021, p. 273 ff.). Placing the emphasis on land imaginaries within processes of land transformation recognizes that economic, financial, or political interests of actors do not directly result in, or translate into, particular outcomes, such as farmland’s financialization, assetization, or digitization. Rather, such projects are fed by certain worldviews or ideologies, as well as associated ideas, visions, hopes, and dreams regarding land. Land imaginaries can take shape as rather implicit, subconscious, and underlying understandings of land as well as explicit and conscious expressions of specific ideas or purposes surrounding land. Within the latter, imagining is seen not only as a mental function but also as a social practice of actively envisioning and ‘bringing into being’ new worlds and different realities. This active dimension is especially prominent in Sheila Jasanoff’s concept of ‘sociotechnical imaginaries’, which, linking past, present, and future, emphasizes the aspiration for social change implicated in the notion of imaginaries (Jasanoff 2015). With regard to this paper’s focus on the active making of land’s financialized future, I am especially interested in exploring this world-making function of land imaginaries as actively envisioned expressions or pathways to realize this financial future.

Lastly, insights from the interdisciplinary field of critical data studies, and recent work at the intersection of critical data and environmental studies, are valuable to complement the perspectives outlined so far. Exploring the significance and power of digital data in contemporary society along with the role that data play for societal transformation (Hepp et al. 2022, p. 6), critical data studies pursue the overall goal of problematizing inherent assumptions about data and politicizing big data in a context where positivistic approaches to data prevail (Iliadis and Russo 2016). One key sociotechnical transformation in regard to data is the turning of social action and processes into ‘quantified data’, allowing for real-time tracking and predictive analysis (Hepp et al. 2022, p. 5). Closely intertwined with digital technologies, this ‘datafication’ never occurs as a ‘1:1 representation’ of people and their practices, but, similar to the imaginaries and models discussed above, it is always embedded within complex interactions and the pursuit of certain purposes. In other words, data are never neutral, but interest-driven technical articulations: ‘[d]ata do not provide a window on the social world and represent independently existing phenomena, the relationship with the social world they are meant to represent is recursive’ (Hepp et al. 2022, p. 5).

Data are moreover informed by specific histories, ideologies, and philosophies, which often remain hidden (Iliadis and Russo 2016). In ‘Western’, i.e. European and North American cultural contexts, numbers assume a specific prominence and status. They are perceived as raw, objective, and neutral, and granted special authority, as especially ‘credible’ representations of the world (Espeland and Stevens 2008, p. 416–417). As Iliadis and Russo elaborate:

‘[Numbers] are couched in a rhetoric of factuality, imbued with an ethos of neutrality, and presented with an aura of certainty. They step out of the shadows of their human creators, enter center stage, and, in the arguments and claims of countless profiteers, start to speak for themselves’ (Iliadis and Russo 2016, p. 4).

Assumptions regarding accuracy and objectivity are particularly present in the discourse around big data (boyd and Crawford 2012; Kitchin 2014), and new ‘smart’ digital technologies, including those currently being developed for environmental management (Gabrys 2016, 2020; Nost and Goldstein 2022) and farming (Bronson 2022; Carolan 2020; Fraser 2019; Miles 2019; Klauser and Pauschinger 2022; Taiuru et al. 2022). One objective of critical data studies is to question this aura of objectivity and certainty and to expose ‘the societal embeddedness and constructedness of data’ (Richterich 2018, p. 2), by providing a detailed description of people’s data practices (Hepp et al. 2022, p. 7). In a similar vein, recent scholarship on environmental data investigates specifically how different environments are increasingly becoming ‘technologized sites of data collection, processing, and analysis’ (Gabrys 2020, p. 1), and how this includes ‘particular ways of materializing environments and ways of acting on environmental problems’ (Gabrys 2016, p. 2). In addition to data practices, such as observing, measuring and monitoring, processes of automation, and forms of governance embedded in and enacted by data, this literature has emphasized the materiality of data technologies themselves by engaging the notion of ‘data infrastructures’ (Nost and Goldstein 2022; Goldstein and Nost 2022). Data infrastructures, as Nost and Goldstein stress, are ‘socio-material’, they include the ‘place and time-specific networks of funding, standards, rules, technologies, and environments’ (Nost and Goldstein 2022, p. 5) as well as situating data materially ‘in terms of where, and from what, they are derived as well as what, and whose, natures they imprint’ (Nost and Goldstein 2022, p. 7). The notion of ‘data infrastructure’ will thus be useful to emphasize the interplay between immaterial and material components within farmland investments.

Below, I will use the combined conceptual lenses outlined in this section to examine how farmland investment brokers perceive, construct, and engage with risks and data surrounding farming, and translate these into ‘land imaginaries’ that help render farmland investable for their investors. I then turn to the more material practices of putting the necessary data infrastructures in place to collect and capture the required farm data. This is followed by a number of suggestions for important questions emerging out of this work that future research should address.

## Controlling farming: imagining the ‘farm as a factory’

In the farmland investment discourse, farmland investments have been touted as safe and secure investments, referring to Mark Twain and his alleged recommendation ‘buy land, they are not making it anymore’ or by depicting farmland as ‘gold with yield’ (Fairbairn 2014; Visser 2017). In practice, however, the Australian farmland investment brokers I interviewed during my research had to work hard to attract financial investors to this new investment space. One component of this work was ‘imaginative work’ in the form of storytelling. Storytelling is not just a subjective, individual activity, but embedded within relational practices (Fairbairn et al. 2022). It is intersubjective and context specific as ‘[a]ctors construct stories deliberately and for specific reasons’ (Birch 2023, p. 31). Farmland brokers, I suggest below, do not come up with imaginaries of investable farming on their own, but develop them in a co-creative process.

Throughout my research, one of the key challenges my informants encountered in making farmland investments attractive for their investors was the volatility and unpredictability of farming, and the sense of lacking control that comes with it. As Stuart, a farmland investment broker who was working with an international endowment fund, told me, a sense of lacking control combined with a ‘misunderstanding’ of the risks involved in agriculture scared a lot of financial investors away:

‘They [financial investors] love the idea of farming and exposure to agriculture, but when it comes down to it they just don’t understand the risk that they’re taking on. The volatility of farming. Then as soon as you hit a drought, people freak out and they want to sell’ (Stuart, farmland investment broker, 2017).

The challenge my informants encountered, specifically in raising institutional capital, was to explain to financial investors what farming actually ‘is’, and to give investors an understanding of how agriculture behaves as an asset class. As Stuart told me, ‘you’ve got to understand the asset class. Understand what you’re doing, and have a mindset – you have to sit back and wait, but be ready when the opportunity presents itself’ (Stuart, 2017). In his experience, however, sitting back and waiting was ‘a hard thing’ especially for corporate investors, as they did not necessarily understand that farming was ‘a different asset class to play in’ (Stuart, 2017). Faced with this situation, farmland investment brokers often told me that they saw the need to ‘educate’ investors on the specifics of agriculture, and how it behaved as an asset class:

‘[Interest in agriculture] is growing. We’re starting to see some. But it’s slow. It’s about that education process. They don’t really understand what it is they’re investing in’ (farmland investment broker, 2016).

But what is the farm-based asset class that financial investors are investing in? I suggest that investors are not investing in some kind of ‘pre-existing’ farming that is already there, but rather that it is the interest in farming from the financial sector combined with the work of farmland investment brokers that leads to a new kind of ‘investment ready’ farming emerging. In other words, ‘investment grade assets’ are not ‘out there’, but are being ‘co-produced’ (Jasanoff 2004) between financial investors and farm investment brokers in order to align the investment logics of the financial sector with the capital interests of productivist agriculture. Rather than being a ‘one way road’, the ‘education’ of investors about farming as an asset class is a co-constructive interaction between financial investors and investment brokers, where investors are ‘educated’ on farming just as much as investment brokers are learning how to produce investor-suitable imaginaries of farming.

The following account provided by Andrew, a farmland investment broker with an extensive background in corporate farming and asset management, illustrates this co-construction. He draws on the prominent imaginary of the ‘farm as a factory’ (Fitzgerald 2003) to explain to his prospective clients what it is they would invest in:

‘So say for a beef farm, what we do in a breeding farm is we’ve got cows and that’s like a factory. We’ve got bulls and they come in. They’re just an input to the factory. Then we end up with baby cows that we sell. So from a factory point of view it’s pretty easy to understand. We buy young animals, we grow them for a while, and then we sell them. So that’s a bit more like a nursery or something that gets little seedlings, grows a flower and sells the flower. So that’s a bit more like it. So once you start to take them through that they get it’ (Andrew, farmland investment broker, 2016).

Whether or not Andrew indeed believed in this ‘factory style farming’ himself, is less important, I suggest, than what this imaginary does, or helps him to do. The imaginary of the ‘farm as a factory’ starts from a point of familiarity, the factory as something that the investor understands and assumably appreciates. This imaginary further invokes notions of the ‘modern’ form of agriculture that emerged in the US after the first World War, and which was modeled upon factories and business enterprises, including characteristics such as ‘[t]imeliness of operations, large-scale production sites, mechanization, standardization of product, specialization, speed of throughput, routinization of the workforce, and a belief that success was based first and foremost upon a notion of “efficiency”’ (Fitzgerald 2003, p. 5). All of these, as Deborah Fitzgerald writes, were essentially built upon ideas of technological and scientific innovations and the spirit of rationalism.

While some agricultural systems, such as the beef production Andrew referred to, are indeed closer to the controlled environment of a factory than others, it is also clear that the majority of farming is still far away from taking place under such ‘factory like’ conditions. It is no coincidence that David, quoted in the introductory vignette, used the imaginary of the factory to depict exactly the opposite of farming. Regardless of this reality, however, the imaginary of the farm as a factory embodies the ‘ideal’ kind of farming that the investor would want to believe and invest in. Presenting farming as such a ‘factory-like’ activity allows Andrew to convey to his investors a notion of control, suggesting that farming takes place in the ordered, structured, and regulated environment of a factory, where there are limited variables and predictable outcomes, and therefore limited risks. Andrew, I suggest, uses this imaginary in a conscious and reflexive way, it becomes a ‘knowledge claim’ about the world that helps him within his performative ambition ‘to shape the world’ into specific directions (Birch 2023, p. 34/38).

It goes without saying that the imaginary of ‘controllable farming’ alone is not sufficient to establish an investment grade asset. As I will argue below, the imaginary of the factory farm is being complemented and sustained with additional farmland imaginaries expressed in calculative practices, which reimagine farming within the language of numbers. These numbers, the ‘hard facts’ of data, are supposed to further help render the farm like any other factory, real estate, or infrastructure investment by putting a set of quantifiable metrics around farming. Complementing the story-based re-imagining of the farm illustrated above, these metrics are intended to create the sense of ‘certainty’ for investors that suggests control and predictability. But what kind of data are required here, and where do they come from?

## Measuring farming: the ‘lack of data’

During my research I learned that, in addition to the volatility, unpredictability, and lack of control around farming, my informants identified a ‘lack of data’ as another major barrier for the placement of financial capital in farming. In comparison to other investment opportunities, I was frequently told, there were few data available for investors to assess farmland investments:

‘Data and history are important to investors. That’s where investors struggle. Let’s be honest there, why do you think there aren’t a lot of investors in agriculture? It’s because the data is not there. That’s a big gap. Investors like … — when they go and invest in the stock market, they can see what an index has done for years and years and years. It’s very accessible data when you start dealing with tradable securities and what not. This is agriculture, which is not as sophisticated so to speak when it comes to that’ (farmland investment broker, 2017).

The main reason farmland investment brokers identified for this situation is that much of Australian farming is still run by ‘mom and dad’ farmers. As Tom, who had worked in farmland investment in the US for several years, told me, ‘in Australia you buy a farm today and the agent might go yeah, there’s 3,000 acres out there and that’s it. There’s no measurement of that, there’s no surveying of it’ (Tom, 2017). In comparison to his experience in the US, he found that there is very little information available for farms in Australia. While in the US, he could access extensive data sets on arable land, soil maps, and yield data from the USDA or insurance schemes, in Australia ‘it’s a little bit of a smokes and mirror when it comes to agriculture information because the farmers in Australia are a lot more diverse’ (Tom, 2017). In his view, Australia is a rather ‘unsophisticated market’ in regard to farm level data.

Digging deeper into this data situation, however, it became apparent that the ‘lack of data’ is not just a question of available data, but it is the origin, production, and delivery of data that is important. As it has been well established in critical data studies, some data are regarded as more reliable and accurate than other data (boyd and Crawford 2012; Iliadis and Russo 2016; Kitchin 2014). In the same interview, while complaining about the lack of data in Australia, Tom also told me that a lot of the information his company was looking for when considering the purchase of a farm was ‘not rocket science’. For instance, this could be simple things such as the fertilizer and chemical application on a paddock. A lot of farmers had this information ‘either in their head or they have it written on the back of an envelope or in a diary or whatever it might be, or maybe they have a computer system with a bit of that information’ (Tom, 2017). But this kind of data and the form of the data delivery are not necessarily considered trustworthy:

‘How accurate is that really? At the end of the day someone can hand you a paper that’s been written and yeah, we got two tons that year and three tons that year and whatever. Is that really accurate? Are you really going to take that as gospel, true, or is that just someone writing something down because they felt like it?’ (Tom, farmland investment broker, 2017).

Partly, this lack of trust in farmer information is due to family farmers’ assumed reporting practices. As interviewees repeatedly suggested during my research, farmers’ financial statements are seen as geared towards minimizing tax payments, as ‘a lot of family farms for taxation reasons, [t]hey’re not necessarily recording things accurately’ (farmland investment broker, 2016). These assumptions regarding farmers’ practices and a general mistrust of the information received from farmers, however, combine with further understandings as to what kinds of procedures produce the ‘legitimate’ and ‘accurate’ data suitable to draw conclusions surrounding investment decisions. This became clear when Andrew told me that he, as a general rule, just ignores what the vendor tells him:

‘For me, I’ve got to actually make a lot of assumptions. The vendor might say, oh yes, I’ve got five tons, five tons, five tons. We’d have to discount that. We’d have to calculate what we think. *Because it’s not audited*, and therefore you have to take it with a grain of salt’ (farmland investment broker, 2016; own emphasis).

When speaking of data, farmland investment brokers are thus not only looking for any kind of ‘data’ on, or numerical representations of, farming, but the data requested need to have a certain credibility – they need to be ‘audited’. For Andrew this lack of audited data also meant that he was taking on greater risks when investing in a farm, so he would offer a lower price. The ‘lack of data’ thus translates into higher risks (a point I will come back to in more detail below). Personal communications or the kind of bookkeeping that family farmers might have undertaken do not grant data credibility, but data credibility is rooted in the specific form of producing quantifiable knowledge about the world that has been conceptualized as ‘audit culture’ (Shore and Wright 2015; Strathern 2000). Going beyond the use of numerical indicators and rankings as a key element of contemporary governance, audit culture refers to the increasing application of the principles and practices of modern accounting and financial control, and the widespread proliferation of these calculative rationalities, in contexts far removed from the world of bookkeeping and corporate management (Shore and Wright 2015, p. 421–422). In short, when Tom told me that for him data was what ‘the investors really want to know’ (2017), he was referring to a particular kind of data that are produced according to those specific financial accounting practices, which endow data with the credibility and trustworthiness they need in order to be acknowledged by investors.

## Calculating farming: missing pasts, modeling futures

Faced with the lack of ‘suitable investment data’ on the past, farmland investment brokers try and calculate the future. One calculative technique that is used here is a simple form of modeling.^5^ Based on environmental data in combination with assumptions made on the costs of inputs as well as market developments, farmland investment brokers develop farm specific models to calculate the potential returns from an asset. Two examples illustrate how my informants are feeding and using these models, and thereby further illuminate their engagement with, and perception of, data. In the following interview quote, Andrew explained to me, how his company models the prospective output of a grain farm:

‘So say for a grain property we know what the price of fertilizer is. We know roughly how much fertilizer we need to use. We can do some quite robust mathematical formulas around what the average rain is. On that soil type we can then reasonably generate how many kilograms of grain we’ll get. So all of that stuff is pretty robust. [W]e then say right, this farm will generate 3.5 tons per hectare. We can model that out. We’ll need a tractor. We’ll need a utility, a motor bike. It’s all of that the model allows us to very quickly change. [A] lot of the farms are relatively similar. It’s only the size that’s different. So we can go and change all that’ (Andrew, farmland investment broker, 2016).

Another farmland investment company I interviewed also worked with models, in this case with a model developed by the Australian research organization CSIRO (Commonwealth Scientific and Industrial Research Organisation). This model, as my interviewee Jack explained, allowed them to model the future yield in a certain region based on 120 years of historical weather information:

‘Often when we buy a farm, we’ll actually use that model, where you put in the soil type, all the weather data, crop rotations et cetera, and you can then simulate wheat yields for 120 years’ (Jack, farmland investment broker, 2016).

Depending on the proximity of the farm to the weather station, where the weather data are measured, Jack further explained, they either worked with the data received from that specific station, or, if the farm was in between two or more weather stations, the weather data could be interpolated.

When developing models, modelers make decisions as to what variables are included, what data are being collected, and where the knowledge used for the model is coming from (Loconto and Rajão 2020). Importantly, choices made about what is represented and what is left out are not just technical decisions, but ‘normative value judgement[s] signifying that what is made invisible is irrelevant or worthless’ (Loconto and Rajão 2020, p. 2). In this sense, models have the power to visibilize as much as invisibilize. Rather than being neutral devices or simple representations, land models co-produce and perform certain realities of the world, they are ‘world making practices’ (Loconto and Rajão 2020, p. 3). As the above examples demonstrate, farmland investment companies’ models draw on data received from public sources (e.g. on rainfall) and combine these with assumptions (e.g. regarding market prices) and mathematical operations (e.g. interpolation of data). The data used to feed these models reveal what kind of data are considered ‘accurate’ and ‘legitimate’ – namely supposedly ‘neutral’ data received from external, public sources as well as data grounded in and obtained from mathematical calculations. Again, and despite the purpose of the model to be farm specific, personal, or individual, information on the farm remains excluded from the model. The calculative outcome of the model is presented as representing simple, straight forward, and ‘robust’ data, which stands in strong contrast to the information provided by farmers portrayed as ‘unreliable’ and ‘anecdotal’.

Faced with uncertain futures, calculative devices such as models also play an important role in justifying and legitimizing certain actions as they provide scenarios, and therefore calculative grounds, for decision making, while proving that ‘due diligence’ has been performed with regard to possible risks (Beckert and Bronk 2018, p. 18). In this way, models help to envisage the future as a range of alternative scenarios from which investors can choose (Doganova 2018), and thereby ‘“transform” (perceived) uncertainty into (perceived) risk’ (Besedovsky 2018, p. 241). This performative function of models is an important part of farmland investment brokers’ practices, for example for advising investors on investment decisions. As Jack explained to me, the model helps him to explore the investment preferences of his clients, and to understand, for instance, how these preferences translate into their engagement with weather volatility in different regions. Volatility in itself, he said, was not necessarily a bad thing, but always depended on the risk profile of the investor. Institutional investors, who had to report about returns on a regular basis, were more concerned about yearly variation than individuals who were committing their money for twenty or more years, and could therefore ‘just take the ups with the downs’ (Jack, 2016). Based on the model, Jack, together with his investors, identifies how much volatility they will accept, and then makes decisions about investment locations on that basis.

Despite the presentation of the modeling data as ‘robust’ and ‘reliable’, I found that uncertainties remained, most notably with regard to the possible implications of climate change. How do farmland investment brokers deal with these fundamental uncertainties? As Australia is particularly exposed to climate change, I tried to understand how my informants include considerations about climate change in their models, given that the ‘accurate’ weather data they relied on for their models is inevitably data derived from the past and, as such – and especially with the prospect of potentially rapidly changing weather conditions – has limited relevance for the future. However, for different reasons, possible effects of climate change were not considered in my informants’ models. For Andrew, the implications of climate change were simply too long term, so that he did not consider them relevant for his clients:

‘Over 50 years we may see an increase in temperature of, say one degree, which may have an impact in certain areas. Fifty years, it won’t be this particular set of investors that we’re worrying about. In fact it won’t be me. We’re aware of it but we think the trends are more long term. As a globe, yes, we should be interested in it, but as a particular investor today the impacts are pretty light’ (Andrew, farmland investment broker, 2016).

Instead, the company’s focus was on trying to mitigate the effects of climate variability by managing variability geographically across Australia. This kind of geographical diversification is a common strategy applied in farmland investment (Fairbairn 2020, p. 83 ff.). Jack, on the other hand, had tried to include climate change factors in his models, but, as he told me, was faced with the limits of modeling climate change impact:

‘The trouble is, we don’t have data for future variability. We know that it’s probably going to be more variable. But to what extent, and all that sort of stuff is very hard to quantify. Particularly, you see maps of changes in rainfall or temperatures for Australia. But if you then say to those climate modelers okay, tell me where I should and shouldn’t invest? So what’s going to suffer from much greater volatility and what are the better places to be? They say it’s hard to build a model that’s accurate on the continental scale. If you ask me on a regional or local scale, the models are just nowhere near accurate enough to be able … — so we’ve asked exactly that question. Can the model say that this area is going to suffer more than this area? No one is game to do that’ (Jack, farmland investment broker, 2016).

His conclusion was that ‘other than sort of say this part might get a bit wet in summer and this part might get a bit wetter or dryer in summer’ the implications of climate change were impossible to predict, and certainly not precise enough to say to someone ‘don’t put your money there, put your money here’ (Jack, 2016). Contrary to Andrew, Jack nevertheless takes climate change into account when making investment decisions. One strategy is to choose soils that are capable of storing moisture, which represents an advantage in situations of more variable climate and more intense rainfall. A second strategy is to pick regions with higher rainfalls than needed, so that in the case of decreasing rainfall it will still be enough. In terms of the concrete modeling practices, however, this does not change the fact that considerations surrounding one of the most, if not the most, profound factors influencing future weather data are not included in the calculations performed by the model.

In sum, as farmland investment brokers struggle with finding historical data at the farm level that fulfills their investors’ requirements, they are filling in the missing past with calculated future scenarios. These future scenarios produce highly selective representations of farms, which model the productive future of a farm based on assumingly neutral data derived from ‘generative’ public data bases, assumptions, and calculative operations, while excluding farm specific ‘local’ knowledge of farmers. At the same time, they also systematically ignore significant factors, such as climate change due to either their perceived irrelevance or the inherent limitations of modeling capacities at the requested scale.

## Re-engineering farming: implementing and commodifying ‘data infrastructures’

While the data situation outlined above is what my informants currently rely on and have to present to their investors ‘because there’s nothing else’, farmland investment brokers pursue the objective to ‘take that to another level’ and build the ‘sophisticated database’ that investors want to see (farmland investment broker, 2017). This request for data was echoed by the investors I spoke with during my research. As one representative of an Australian superannuation fund told me, ‘there needs to be more data from a risk-return standpoint [and there] needs to be more data on a business-by-business level’ (superannuation fund representative, 2017). To see more superannuation fund investment in agriculture, in his opinion, would require higher levels of aggregation and corporatization of farms. Instead of ‘mom and dad’ operating the farm, someone would first need to aggregate these farms, create a more corporate structure, and establish more sophisticated financial reporting:

‘Then an investor could come in and actually look back over the past few years, see what the financial performance was like and better assess risk and return, have more confidence in their underwriting assumptions, price it more aggressively, etc. So that is 100% probably the biggest thing to drive further investment I think is just having more comfort around data’ (superannuation fund representative, 2017).

Given that the levels of record keeping are still comparatively low in Australian agriculture, as outlined above, the challenge for my informants is to build these databases over a short amount of time. This is where digital farming technologies come into play, as they are seen as the tool of choice to produce the kind of ‘accurate’ and ‘neutral’ data requested. Environmental practices, as William Cronon and James Scott have shown, not only ‘measure’ what is ‘there’, but these practices are also transformative as they (make actors) both ‘re-view’ and alter the materiality of nature itself (Cronon 1991; Scott 1998). In a similar vein, the request of financial investors for certain kinds of farm data changes not only farmland imaginaries and calculative practices surrounding farming, but also the very materiality of farms, which are becoming ‘re-engineered’ with digital devices and precision agriculture tools – the material elements of data infrastructures (Nost and Goldstein 2022) – into ‘smart farms’ to respond to this new quest for capturing farm data and building farming track records.

Hugh, a farmland investment broker whose business model it is to develop properties into ‘investment-grade assets’ for institutional investment pointed to this connection in very clear terms. When assessing a farm’s potential to become ‘investable’ for institutional investors, he explained to me, he is looking for a number of features, such as quality natural resources, commodity specialization, appreciable scale, and proven on-the-ground management. However, to become an investment grade asset, the farm first and foremost needs to have a financial track record; as Hugh put it: ‘[We] recognize data as a key asset of a property’ (2019). Currently, however, ‘you start it from a low base’ (2019). The company had not farmed the land for three generations, ‘so we don’t have the wisdom that, oh, that black country always gets wet in a wet year, and the red countries along it perform’ (Hugh, 2019). Equipping the farm with digital farming technologies is thus a key component within the company’s property development plan to implement the material infrastructure required to produce the kind of farm data required for the farm’s assetization:

‘[W]e use precision agriculture as one method, whether it be satellite imagery, EM surveys, etc., etc., to try and build up corporate knowledge of an asset. [W]e need to try and quantify that as quickly as possible and build up that knowledge. So land capacity studies, remote sensing, etc., are a key component of doing so and then putting data capture systems in place. So whether it be variable rate technology, whether it be paddock records for all the agronomy activities, financial records, etc., etc.’ (Hugh, farmland investment broker, 2019).

As the following interview quote demonstrates, beyond the production of data on individual farms, farmland investment companies also try to establish data infrastructures across their different investment geographies to build globally comparable databases:

‘We’re developing some systems in-house that allow us to centralize data, be able to have a one stop shop, go into the mapping system and click on things, and see information and have it all come to it. [W]e are trying to put systems in place that give us a level of consistency as well. Not only domestically but also globally’ (farmland investment broker, 2017).

These efforts put into implementing data infrastructures at the farm level show that ‘investment-grade assets’ can have a variety of material features – but these are of little value for investors if they are not ‘legible’ within the language of investment. Only if the materiality of the farm is represented within the metrical language of finance it becomes amenable for commensuration as ‘the valuation or measuring of different objects with a common metric’ (Espeland and Stevens 2008, p. 408).

In light of the limited availability of audited track records of farm data, farmland investment brokers have identified this commensurability as a scarce resource – and therefore as an additional possibility to generate profit. Data, then, not only represent a key component within the assetization of a property, they are also considered a commodity, as Andrew pointed out:

‘What they [the investors] like to see is, okay well 10 years ago we had this fund. It returned this. Now with this fund it returned that. In agriculture it’s a very new space in terms of institutional management. Therefore anyone with a good track record – it’s a good commodity’ (Andrew, farmland investment broker, 2016).

In 2016, Andrew’s investment company was at a stage where they had raised money for their fund, acquired properties, and started running the farms. Like Hugh, they were aiming to build up a track record for their farms to realize a greater flow of capital, because Andrew (as mentioned above) considered the current lack of ‘accurate’ data as a higher investment risk. If he could be certain about the accuracy of the data, Andrew stated, he would pay more for a property, given the resulting lower investment risk. Hence, his investment strategy was not only based on realizing returns from running and selling the farm, but also on generating additional profit from the data collected over this period of time:

‘[W]hat we think is, when we sell this business, we’ll actually be selling ten years of audited, accurate data. So for anyone buying these farms we’ll take a lot of risk out. [W]hen we sell the farms hopefully we’ll – well, we will have ten years of audited, very robust data that the purchaser can then rely on’ (Andrew, farmland investment broker, 2016).

These quotes illustrate that rather than representing the often touted ‘agricultural revolution’ (Duncan et al. 2021), digital farming technologies are better understood as a continuation of past processes, centered around agricultural intensification, rationalization, and control (Miles 2019). As such, with Christopher Miles, the use of digital farming technologies within farmland’s assetization can be seen as ‘a movement to further transform objects (and now activities) into discrete commodities, to extend the reach of capital, and to accumulate entire new geographies of possibility to the market’s logic’ (Miles 2019, p. 6).

In sum, turning farms into financial assets means that farmland and farming are not only being re-imagined, but these new imaginaries are also complemented and supported by the material transformation of the farms themselves. As Goldstein and Nost emphasize, as our relationships with nature become increasingly digital, this does not mean they become immaterial (Goldstein and Nost 2022, p. 4). Rather, these digital relationships have their very own, although often hidden, materiality (Pickren 2018, 2022). As this section has shown, the material transformation of farms that occurs as part of their assetization first and foremost includes the implementation of comprehensive data capture systems – digital and increasingly ‘smart’ farming technologies that are designed to capture, collect, and compile large amounts of farm data that make farming accessible for risk-return calculations and commensurable with other assets.

## Discussion and conclusion

This paper started from the observation that also in times of increasingly financialized capitalism, the biological factors and ‘stubborn materiality’ of farming are still posing a challenge to integrating agriculture into capitalism. As farmland investment broker David expressed it in the introductory vignette, farming is a dynamic system, it is in a constant state of flux and continuously interacts with manifold other biological and environmental systems. Isolating agriculture from these interdependencies and re-creating it in a ‘controlled’ environment is difficult if not impossible to achieve. As David put it, humans can have some influence – but they can hardly gain full control over agriculture’s destiny. Yet, it is precisely this notion of control that financial investors are looking for when making investment decisions.

Scholarship on farmland investment has only recently started to more closely engage these material dimensions within the financialization of agri-food. Adding to this literature, this paper has focused on the role of farmland investment brokers as intermediaries between the desire of finance to be in a (perceived) position of control and calculable risks, and the practical challenges of navigating the material realities of farming. I have argued that farmland investment brokers do not act on their own but co-create ‘investment grade assets’ with financial investors in a co-constitutive effort to align the investment logics of finance with the capital needs of productivist agriculture. This co-creation of investment grade assets, I have argued, involves both immaterial and material components. Farmland investment brokers co-produce investor-suitable farmland imaginaries that are fed by narrative elements and storytelling, as well as calculative practices of providing (what is perceived and constructed as) ‘accurate’ data and farmland return scenarios. These rather ‘immaterial’ practices are closely linked with more material activities of modeling and data collection, as well as concrete material changes made to farms on the ground. The latter are specifically geared towards implementing data capture systems, which transform farms into ‘smart farms’ equipped with precision agriculture tools and digital devices – that is, data infrastructures that make nature legible for capital. Processes of assetization and digitization of farming are thus not only increasingly intertwined but also mutually condition and reinforce one another.

A number of questions arise from the material presented in this paper that deserve further attention in future research at the intersection of assetization and digitization of agri-food. Taking inspiration from Goldstein and Nost’s outline for a ‘political ecology of data’, I want to sketch these out by engaging the four lenses - political economy, knowledge production, context, and materiality - that they identify as core themes for such a research program (Nost and Goldstein 2022, p. 6 ff.). A political economy perspective points to the dimensions of power that are also involved in making decisions about data production, distribution, and extraction. It is thus crucial to understand who produces, accesses, stores, and controls data, and for what purpose (Bronson 2022; Carolan 2020; Duncan et al. 2022; Fraser 2019). As this paper has shown, the availability of farm data represents a core component within the assetization of farming, currently advanced by financial investors and farmland investment companies. This paper specifically illuminates the intimate connection between farmland assetization and digitization, and corroborates Duncan et al.’s (2022, p. 233/236) recent findings that digital farming technologies are being used as a means of ‘disciplining land’, that is promoted as a key tool for investors to gain control over agriculture’s biological vagaries and uncertainties. It will be important to further investigate how and to what extent farmland investors and their intermediaries are beginning to amass and control large quantities of data that themselves become valuable assets – and are likely to accelerate financialization.

What is more, financial actors not only advance the production and control of farm data, they also increasingly determine what kinds of farm data, farming imaginaries, and agricultural knowledge are being produced, and what types of data are valued over others. Such practices of imagining, producing, and valuing knowledge are never neutral or innocent. Rather, attention to knowledge production allows one to question *whose* truths are made possible through data infrastructures (Lin 2022, p. 286). As this paper has argued, the rendering of farmland as a financial asset involves a profound shift in how land and farming are being re-imagined to become ‘legible’ and ‘commensurable’ within the calculative, number-based, and risk-return focused logics of finance. This means that the production of farming knowledge becomes increasingly influenced by land imaginaries and numeric representations of farms that prioritize the calculation and assessment of investment risks and return opportunities over other forms of farm knowledge. In future research, it will be important to address whose farming imaginations and productions of agricultural knowledge are becoming more powerful – and potentially dominant – and which ones are becoming rather relegated or silenced. This does not only concern forms of Indigenous and non-Western knowledge that have been silenced, and in some cases been made extinct, over centuries (Goldman et al. 2011; Liboiron 2021). As this paper has demonstrated, financial investors and farmland investment brokers in Australia also see the farm data provided by ‘mom and dad’ run family farms as inadequate to determine risk and return profiles. Thus, even in already industrialized farming contexts such as Australia, the co-production of investable farms might further the consolidation and establishment of large-scale, corporate run farms. How the quest for farm data and specific forms of agricultural knowledge will influence and reshape the Australian farming landscape requires further investigation.

What is more, datafication and increasingly numerical farm governance are not restricted to industrialized contexts such as Australia but can just as well apply to farmland investment endeavors in the ‘South’. For instance, also the manager of the finance-backed grain farm in northern Tanzania that Ouma investigated was largely occupied with ‘producing numbers for investors’ (Ouma 2020, p. 139). Such increasing similarities within the universe of global farmland investment, along with still remaining context-specific differences, will need careful empirical investigation as the ‘digital revolution’ is exported to farmers in the Global South (Fairbairn and Kish 2022). As Nost and Goldstein (2022, p. 7) emphasize, despite the seemingly placeless, universal, and ahistorical world of digital data, context still matters – further studies of how farmland assetization and digitization will intersect and combine in the future will need to include more diverse contexts across ‘North’ and ‘South’ geographies.

This brings me to the fourth and final component of the political ecology of data, namely the often-overlooked materiality of data infrastructures. As Pickren (2022) stresses, material data infrastructures are crucial to the circulation and production of liquid natural capital – processes of financialization not only consist of algorithms and code, they also and crucially rely on material infrastructures, such as data centers, fiber-optic cables, or radio towers. This ‘dialectic between moments of abstraction and moments of fixity’ (Pickren 2022, p. 33) also plays out within the assetization and digitization of farmland. As this paper has demonstrated, the translational processes, which make farming accessible within the language of numbers, are both advanced by, and simultaneously advance, the re-engineering of farming materialities on the ground. The request of financial investors for specific kinds of farm data involves profound material transformations of farms which require further attention in agri-food studies. How this material data infrastructure evolves, which farms it will cover and which ones it will spare, how it will connect and intersect with other data infrastructures, and whether it will hold its promises of precision and accuracy are important questions that will need to be addressed in future research.

## Footnotes

1 All names used for interviewees in this paper are pseudonyms.

2 Jasanoff suggests the concept of ‘co-production’ to understand knowledge and its material embodiments as both products of social work and constitutive of forms of social life (Jasanoff 2004, p. 2). While Jasanoff’s focus is on scientific knowledge in particular, I suggest that the insight that ways of knowing the world are inseparably linked to the ways in which people seek to organize and control the world, can be fruitfully extended to knowledge production more generally.

3 Some of these assumptions have proven more uncertain and contested than investors might have anticipated. For an in-depth discussion of the contradictions of farmland investments in Australia see Sippel (2018) and Sippel and Weldon (2021). For a comprehensive mapping of foreign purchases of Australian farmland between 2008 and 2020 see Smith et al. (2022).

4 The concept of ‘financialization’ has become a ubiquitous focus within processes of social and economic transformation (cf. Mader et al. 2020). Financialization and assetization are closely intertwined, as one important way that financialization occurs is through the capitalization of assets (Birch and Ward 2022, p. 8).

5 I repeatedly asked my informants what kinds of programs they were using for their models, and the answer was always Excel. As one informant said, his company worked with spreadsheets they had developed over the last fifteen years: ‘There are some complex formulas in there but [t]hey’re not macros or anything. [T]hey are pretty simple, straightforward spreadsheets compared to how complex Excel can get’ (farmland investment broker, 2016).
